# Exposure to Asbestos-Containing Vermiculite Ore and Respiratory Symptoms among Individuals Who Were Children While the Mine Was Active in Libby, Montana

**DOI:** 10.1289/ehp.0901680

**Published:** 2010-03-23

**Authors:** Lisa C. Vinikoor, Theodore C. Larson, Thomas F. Bateson, Linda Birnbaum

**Affiliations:** 1 U.S. Environmental Protection Agency, Research Triangle Park, North Carolina, USA; 2 Agency for Toxic Substances and Disease Registry, Atlanta, Georgia, USA; 3 U.S. Environmental Protection Agency, Washington, DC, USA; 4 National Institute of Environmental Health Sciences, National Institutes of Health, Department of Health and Human Services, Research Triangle Park, North Carolina, USA

**Keywords:** asbestos, children, Libby, Montana, respiratory symptoms, spirometry, vermiculite ore

## Abstract

**Background:**

Libby, Montana, was home to the largest vermiculite ore mine in the United States. The processing, use, and transport of the ore, which was contaminated with amphibole asbestos, led to generalized contamination of the community. The mine closed in 1990.

**Objectives:**

We examined the prevalence of respiratory symptoms in 2000–2001 and their association with history of vermiculite exposure among people who were ≤ 18 years of age when the mine closed.

**Methods:**

Information on respiratory symptoms and exposure history was collected by questionnaire in 2000–2001, at which time participants were 10–29 years old. Logistic regression was used to model the associations between exposures and outcomes adjusted for age, sex, and tobacco smoke exposure.

**Results:**

Of the 1,003 individuals included in the study, 10.8% reported usually having a cough, 14.5% reported experiencing shortness of breath when walking up a slight hill or hurrying on level ground, and 5.9% reported having coughed up bloody phlegm in the past year. These respiratory symptoms were positively associated with frequently handling vermiculite insulation compared with never handling vermiculite insulation. We found no association between vermiculite insulation in the house and respiratory symptoms. Respiratory symptoms were associated with other vermiculite exposures as well, and the number and frequency of these activities showed a positive trend with usually having a cough. We found no association between any of the activities and abnormal spirometry.

**Conclusions:**

These data suggest that residents of Libby, Montana, who were children when the mine closed experienced some respiratory symptoms associated with asbestos-contaminated vermiculite exposure.

Libby is a small community located in the Kootenai River Valley of northwestern Montana approximately 6 miles southwest of Zonolite Mountain, a mountain containing high concentrations of vermiculite ore. The ore was mined beginning in the 1920s by the Zonolite Company and then later by W.R. Grace, which bought the mine in 1963. It is estimated that between 1924 and 1990, when the mine was closed, a total of 5.8 million tons of vermiculite ore was mined and distributed (Kuntz et al. 2009), making Zonolite Mountain the largest vermiculite ore mine in the United States. In addition to the mine, processing, exfoliation, and shipping preparations were performed in Libby. The ore was then used in many products ranging from insulation to soil additives and fertilizers.

The vermiculite ore contained amphibole asbestos. Libby amphibole asbestos was determined to be composed of tremolite, winchite, and richterite (Meeker et al. 2003). The International Agency for Research on Cancer recently concluded that “all forms of asbestos are ‘carcinogenic to humans’” and that “mineral substances (e.g., talc or vermiculite) that contain asbestos should also be regarded as ‘carcinogenic to humans’” (Straif et al. 2009). As with other forms of asbestos, exposure to Libby amphibole asbestos has been shown to negatively affect human health. Occupational studies have shown increased morbidity and mortality (specifically from respiratory cancers and other respiratory diseases, including asbestosis) associated with exposure to the asbestos-contaminated vermiculite from Libby (Amandus and Wheeler 1987; Amandus et al. 1987; McDonald et al. 1986, 2004; Sullivan 2007). Studies have also shown increased morbidity and mortality among individuals living in Libby, Montana, who did not work at the mine [Agency for Toxic Substances and Disease Registry (ATSDR) 2000, 2002; Muravov et al. 2005; Noonan et al. 2006; Peipins et al. 2003; Whitehouse et al. 2008].

Health effects continue to be seen in former workers and community members. In 2000–2001 the ATSDR began a screening program for individuals living and/or working in Libby. Using these data researchers have found positive associations between asbestos-contaminated vermiculite exposure and pleural abnormalities seen by radiograph (Peipins et al. 2003) and self-reported history of systemic autoimmune diseases (Noonan et al. 2006). Despite the mine’s closure, there is concern about the long-term effects of the mining operation for both those exposed in childhood and those exposed to lingering environmental contamination. This age group is of particular importance because lung development continues through adolescence until about 18 years of age in girls and the early 20s in boys. Previous research has shown reduced lung development in children 10–18 years of age who were exposed to ambient air pollution (Bateson and Schwartz 2008; Gauderman et al. 2004). Using the available ATSDR survey data, we examined the prevalence of respiratory symptoms and their association with vermiculite exposure among individuals who were no older than 18 years of age while the mine was in operation and who were continually exposed through lingering environmental contamination. This has not been addressed by previous research.

## Materials and Methods

In 2000 and 2001, ATSDR conducted a study of individuals who resided, worked, attended school, or participated in other activities in the Libby area for at least 6 months before 31 December 1990. In-person interviews and medical tests were performed in 2000 (July–November) and 2001 (July–September). Trained interviewers administered a computer-assisted questionnaire to obtain information on demographics, residential history, household contact with a W.R. Grace employee, occupational history, recreational activity history, history of other potential vermiculite exposures, individual and household members’ smoking status/history, medical history, and current symptoms/illnesses. Parents completed proxy interviews for children < 18 years of age. In addition to interviews, medical tests including spirometry were performed. Radiographs were taken on individuals ≥ 18 years of age at the time of the interview. More than 7,000 current and former community members were interviewed. All participants provided informed consent under a protocol approved by the institutional review board of the Centers for Disease Control and Prevention. Further details on the study can be found elsewhere (Peipins et al. 2003).

For this study we included only those ≤ 18 years of age at the time of the mine’s closure (1990; *n* = 1,224). At the time of the ATSDR study, the age of these individuals ranged from 10 to 29 years. We were interested in studying community-based exposures rather than occupational exposures, so we excluded any individuals that responded they had *a*) worked for W.R. Grace, *b*) worked as a contractor for W.R. Grace, *c*) ever been exposed to dust at other jobs, or *d*) ever been exposed to vermiculite at other jobs (*n* = 221).

In the questionnaire, individuals were asked if they had ever participated in six activities related to vermiculite exposure, including handling vermiculite insulation, participating in recreational activities along Rainy Creek Road (the road leading to the mine that at the time was gravel and was later found to be contaminated with mine tailings), playing at the ball fields (which were located near the expansion plant where there were large piles of vermiculite), playing in or around the vermiculite piles, heating the vermiculite to make it expand/pop, and participating in other activities resulting in contact with vermiculite insulation, products, or ores. Responses to the questions about activities were given as “never,” “sometimes,” or “frequently.” In addition, we created an exposure variable categorizing the combination of the above listed activities into never participating in any of the activities, participating only sometimes in any of the activities, participating frequently in one or two of the activities, and participating frequently in three or more of the activities.

First, we determined the prevalence of cough [defined as “having a cough on most days (at least four days out of the week)”] in our study population. For comparative purposes, we also determined rates of similar respiratory symptoms (cough, defined as “usually cough[ing] on most days for three consecutive months or more during the year”) among individuals of the same age across the United States. This was done using the weighted National Health and Nutrition Examination Survey (NHANES) database from 2001–2002, which corresponds closely to the years of the ATSDR study (Centers for Disease Control and Prevention 2009). The definitions for cough vary slightly between the two databases. Other self-reported respiratory symptoms had greater discrepancies between definitions from the ATSDR survey and NHANES. Therefore, we did not attempt any comparisons beyond that of “usual” cough.

We performed unconditional logistic regression on our study population examining multiple self-reported symptoms (usually having a cough, coughing up bloody phlegm in the past year, being troubled by shortness of breath when walking up a slight hill or when hurrying on level ground, and having a physician-diagnosed lung disease or condition) and responses to the six questions about activities related to potential vermiculite exposure. Information on respiratory symptoms was acquired with yes/no questions included in ATSDR’s questionnaire. We controlled the analyses for sex, age, smoking status (never, former, and current), and family smoking status (living/lived in a household with a smoker, not living/never lived in a household with a smoker). Using likelihood ratio tests to compare all models, effect measure modification was assessed for having lived with an individual while he or she was employed by W.R. Grace. No effect measure modification was seen, so no interaction terms were retained in the final models. This variable was also assessed for confounding using backward elimination with a 10% change-in-estimate criterion.

We ran a second set of logistic regression models with the same exposures but using the results of spirometric testing as the outcome. Spirometry was performed using guidelines of the American Thoracic Society (1995). Results rated as “acceptable” or “suboptimal” were retained but results rated “questionable” or “uninterpretable” were excluded (American Thoracic Society 1995). Predicted values were calculated based on the lower limit of normal (LLN) using published reference equations (Hankinson et al. 1999). The results were classified as normal [forced expiratory volume in 1 sec/forced vital capacity (FEV_1_/FVC) ≥ LLN and FVC ≥ LLN], restrictive (FEV_1_/FVC ≥ LLN and FVC < LLN), obstructive (FEV_1_/FVC < LLN and FVC ≥ LLN), or mixed (FEV_1_/FVC < LLN and FVC < LLN). Because of limitations in sample size, we collapsed these categories to normal and abnormal (restrictive, obstructive, and mixed). Again, we controlled for age, sex, and tobacco exposure (personal and family smoking status). Results of radiographs are not included here because almost half the study population did not have a radiograph and the results on those ≥ 18 years at the time of the ATSDR study in 2000–2001 were reported in a previous study (Peipins et al. 2003).

## Results

A total of 1,224 individuals included in the ATSDR survey were ≤ 18 years of age at the time of the mine closure. At the time of the ATSDR survey, the age range of study participants was 10–29 years. Two hundred twenty-one individuals worked for W.R. Grace, worked as a contractor for W.R. Grace, had been exposed to dust at other jobs, and/or had been exposed to vermiculite at other jobs and were therefore excluded from the study (of these 68.3% were men). The remaining study population contained 1,003 individuals, most of whom were women (57.2%) (Table 1). The data were essentially complete for all participants (see Table 1 note). Most individuals reported never smoking, but 7.3% of study participants stated that they were former smokers, and 15.8% stated that they were current smokers. A small number of individuals had abnormal spirometry results, but other respiratory symptoms, such as usually having a cough or being troubled by shortness of breath when walking up a slight hill or hurrying on ground level, were reported with greater frequency (5.1–14.5%).

Overall the prevalence of usually having a cough was 10.8% [95% confidence interval (CI), 8.9–12.7%] in Libby compared with 5.6% (95% CI, 4.1–7.0%) in the NHANES population. The rates among both populations increased with age; however, in all categories, the rates were higher among Libby residents. The rates in Libby went from 7.3% (95% CI, 5.0–9.6%) among 10- to 16-year-olds to 15.3% (95% CI, 10.3–20.3%) among 23- to 29-year-olds. For the NHANES population, these rates are 2.3% (95% CI, 1.3%–3.4%) and 6.6% (95% CI, 3.2–10.0%), respectively. Also, within each age group, the prevalence of usually having a cough was similar between men and women from Libby; in the NHANES population, the prevalence was generally higher among men. However, the prevalence between the two study populations is not directly comparable because the NHANES questionnaire gives a time frame for having a cough, whereas the ATSDR study simply asked about “having a cough on most days” without specifying the length of time the cough had been prevalent. In addition, the population may differ in other regards, such as smoking prevalence or exposure to different air pollutants.

After adjustment for age, sex, and tobacco exposure, frequently handling vermiculite insulation was positively associated with self-reported cough [odds ratio (OR) = 3.38; 95% CI, 1.56–7.31], shortness of breath (OR = 3.28; 95% CI, 1.63–6.63), and coughing up bloody phlegm (OR = 2.75; 95% CI, 1.07–7.09)] (Table 2). Usually having a cough was also associated with frequently participating in recreational activities along Rainy Creek Road and frequently heating the vermiculite to make it expand/pop. Cough was the only symptom associated with the heating of vermiculite, but frequently participating in recreational activities along Rainy Creek Road was related to shortness of breath (OR = 1.89; 95% CI, 1.12–3.19) in addition to its association with cough. The only respiratory symptom associated with frequently playing at the ball fields near the expansion plant was coughing up bloody phlegm (OR = 2.46; 95% CI, 1.04–5.79). Although some of the point estimates were elevated, none of the activities were clearly associated with self-reported lung disease. We further examined the activities by grouping the number/frequency of activities into four categories: never participating in any of the activities, participating only sometimes in at least one of the activities, participating frequently in one or two of the activities, and participating frequently in at least three of the activities. We found a positive trend between number/frequency of activity and usually having a cough. Compared with those who never participated in any of the activities, individuals who participated in at least one activity sometimes, one or two activities frequently, or at least three activities frequently had ORs of 1.88 (95% CI, 0.71–5.00), 2.00 (95% CI, 0.76–5.28), and 2.93 (95% CI, 0.93–9.25), respectively. We found no association between the number/frequency of activities and shortness of breath, coughing up bloody phlegm, or having a lung disease. The associations present between specific activities and outcomes are similar to those without adjustment for the smoking variables, but the 95% CIs were more precise and the association between number/frequency of activities and usually having a cough did not include the null when we removed the smoking variables from the models. Controlling for only age and sex, the OR for usually having a cough comparing individuals who reported frequently participating in three or more activities and individuals who reported never participating in any of these activities was 3.62 (95% CI, 1.17–11.16).

Handling vermiculite insulation was positively associated with three of four self-reported outcomes we examined. Therefore, we decided to conduct an additional analysis regarding vermiculite insulation present in the house. All individuals were asked if vermiculite insulation was present in any of the Lincoln County addresses at which they reported living (Lincoln County includes Libby, Montana, and surrounding areas). Two hundred seventy-nine individuals reported vermiculite insulation present in the home, 633 reported no vermiculite insulation present, and 91 did not know (these 91 individuals were excluded from this further analysis). Controlling for age and sex, the OR for self-reported coughing up bloody phlegm in the past year comparing individuals with vermiculite insulation present in their house and individuals without vermiculite insulation was 1.58 (95% CI, 0.90–2.76). We also found no association for cough (OR = 1.12; 95% CI, 0.71–1.77), having shortness of breath (OR = 0.95; 95% CI, 0.62–1.45), or having lung disease (OR = 0.65; 95% CI, 0.33–1.30). The results were unchanged when adding personal smoking status and living/lived with a smoker to the model (data not shown). We conducted a sensitivity analysis by reclassifying the study participants with missing information on whether vermiculite insulation was present in their home. When all individuals with missing information were classified as having vermiculite insulation present in the house, the above results were essentially unchanged. The same was true when those with missing information were reclassified as living in homes without vermiculite insulation.

Finally, we examined the association between each of the activities as well as the variable for the combined activities with objective measurements from the spirometry testing (Table 3). We found no association between any of the exposure variables and an abnormal spirometry test result when adjusting for age, sex, personal smoking status, and living/lived with a smoker. The results were very similar when including only age and sex as covariates in the models (data not shown). We also performed an exploratory analysis to see if the association varied between obstructive and restrictive spirometry results (data not shown). Because of small numbers, these CIs were very wide, making it difficult to discern differences. However, it does not appear that the associations varied between the two types of abnormal results.

## Discussion

This study has demonstrated a positive association between certain activities resulting in exposure to asbestos-contaminated vermiculite and self-reported respiratory symptoms among individuals who were ≤ 18 years of age at the time of the mine closure in Libby. Frequent handling of vermiculite insulation was associated with self-reported cough, shortness of breath when walking up a hill or hurrying on level ground, and coughing up bloody phlegm. However, in this study none of these outcomes were associated with simply living in a house with vermiculite insulation. Other exposures, such as frequently participating in recreational activities along Rainy Creek Road and frequently heating the vermiculite to make it expand/pop, were also associated with some adverse self-reported respiratory symptoms. The number/frequency of activities had a positive trend with having a cough. Although the CI was wide when including smoking variables as covariates in the models, the trend with increasing frequency/number of activities remained. The number/frequency of activities was not associated with coughing up bloody phlegm or shortness of breath. None of the exposures were convincingly associated with self-report of having a lung disease or condition. However, because the individuals included in this study are young, it is possible that lung diseases or conditions did not yet have sufficient time to develop. Also, we found no associations between any of the exposures and abnormal spirometry results. Future studies will be important to follow this cohort and assess whether abnormal spirometry results and lung diseases do develop among this cohort of children.

Overall, the rates of usually having a cough were higher among study participants than among the general U.S. population as represented by the NHANES data. Although these results are indicative of adverse respiratory symptoms due to living in the Libby area, it is possible that factors other than exposure to asbestos-contaminated vermiculite contributed to these elevated rates of cough. First, there is the discrepancy in definitions, with NHANES specifying that the cough was present for at least 3 consecutive months, whereas no time limitation was given in the ATSDR study. Second, because of the landscape of the area and the use of wood-burning stoves that occurred until recently in Libby, Montana, it is possible that the higher rates of cough in Libby can be attributed to higher levels of particulate matter in the air. Other studies have demonstrated a positive relationship between levels of particulate matter and cough among children and adolescents (Epton et al. 2008; Pierse et al. 2006; Zhang et al. 2002). For the study years (2000 and 2001), the average annual levels of particulate matter ≤ 2.5 μm in aerodynamic diameter (PM_2.5_) in Lincoln County exceeded the annual standard for PM_2.5_ (standard for PM_2.5_ was an annual mean of 15.0 μg/m^3^; annual mean in Lincoln county was 17.1 μg/m^3^ in 2000 and 16.2 μg/m^3^ in 2001) (U.S. Environmental Protection Agency 2009). However, there is no reason to think that exposure to ambient particulate matter confounds the association seen between vermiculite exposure and respiratory outcomes.

No previous studies have examined exposure to asbestos-containing vermiculite and respiratory symptoms among this age group. There are numerous case reports for individuals with asbestos exposure during childhood and the development of mesothelioma early in adulthood (Arul and Holt 1977; Cazzadori et al. 1992; Inase et al. 1991; Schneider et al. 1996), but only two studies (Andrion et al. 1994; Lieben and Pistawka 1967) have reported on individuals diagnosed with mesothelioma before age 18, which presumably resulted from childhood exposure to asbestos. Our study population had no cases of mesothelioma, but respiratory symptoms were apparent and showed an association with exposure to asbestos-containing vermiculite. Our study is unique in that it explored respiratory symptoms among a group of individuals between the ages of 10 and 29 years. The results are in line with what other researchers have reported for the same study population. As reported by Peipins et al. (2003), there was a positive association between asbestos-containing vermiculite exposure and radiographic abnormalities among adults (≥ 18 years of age). Although Peipins et al. did not examine self-reported respiratory symptoms, they found that respiratory morbidity (i.e., radiographic abnormalities in the form of pleural plaques) was associated with exposure to the vermiculite in Libby.

One of the strengths of this study is that it included a large number of individuals, many of whom had only community-based exposure to vermiculite. Although some individuals had additional exposure through household contact with a W.R. Grace employee, we did not detect any effect measure modification or confounding by this variable. Also, there were very few missing data on the exposures and outcomes of interest. One limitation of the study is that we were not able to determine if the elevated levels of cough were due to asbestos-contaminated vermiculite exposure or to some other differential exposure present while these individuals were children in Libby. Although possible, it is not clear how another exposure, such as particulate matter, could be highly correlated with frequently engaging in activities resulting in exposure to vermiculite. Second, Libby residents are well informed about the contamination in their town and the potential health effects. This may have resulted in recall bias or exaggerated reporting of respiratory symptoms or of exposure frequency. However, we found no association between self-reported physician-diagnosed lung disease and activities related to vermiculite exposure. If individuals were biased in their reporting, we would have expected to see associations between activities with vermiculite exposure and all self-reported respiratory outcomes, although lung disease may be different from the other self-reported outcomes because it is less subjective and possibly less susceptible to recall bias. Exposure assessment involving questions about vermiculite insulation are also subject to recall. Typically, individuals may not be knowledgeable about whether there was vermiculite insulation in any of their childhood residences. In Libby, individuals are acutely aware of vermiculite contamination of their town and households, so it is more likely that they would know what type of insulation was present in their house. However, “don’t know” was a response option and was chosen in similar proportions across all age groups. We performed a sensitivity analysis that included these individuals; the results were essentially unchanged in the sensitivity analysis, demonstrating that the missing data were unlikely to have biased the results. Another limitation is that the participants volunteered to take part in the study and were not a random sample of the population. Also, bloody phlegm is reported as a respiratory outcome in this study, but this variable may not represent health outcomes that are as serious as are usually indicated by this symptom. The most common causes of bloody phlegm in children are not associated with asbestos-related diseases (Pianosi and Al-Sadoon 1996). On the other hand, among adults exposed to asbestos, bloody phlegm would lead to a suspicion of lung cancer, although lung cancer is unlikely in this age group. Finally, the accuracy of the data is based on the reporting by 10- to 29-year-olds, who may not be able to recall with complete confidence all of the exposures and outcomes in question. The data were not linked to any medical records for verification. However, it is unlikely that general inaccuracies in reporting would produce the consistent pattern seen among the prevalence rates for cough and the associations with adverse respiratory symptoms reported here.

## Conclusions

This study was the first to examine the association between asbestos-contaminated vermiculite exposure and the prevalence of respiratory symptoms among individuals no older than 18 years of age while the mine was active and who were continually exposed through lingering contamination in Libby. We found positive associations between some activities likely resulting in asbestos-contaminated vermiculite exposure and certain self-reported respiratory symptoms among Libby residents who were children when the mine closed. The number/frequency of activities was positively associated with self-report of usually having a cough. Frequently handling vermiculite insulation was associated with usually having a cough, having shortness of breath when walking up a hill or hurrying on level ground, and coughing up bloody phlegm. However, the presence of vermiculite insulation in the house was not associated with these outcomes. Other activities associated with self-reported respiratory symptoms were frequently participating in recreational activities along Rainy Creek Road, playing at the ball fields near the expansion plant, and heating the vermiculite to make it expand/pop. No association was detected between any activities with potential asbestos-containing vermiculite exposure and lung disease or abnormal spirometry results. In conclusion, Libby residents who were children when the mine closed experienced some self-reported respiratory symptoms in association with their exposure to asbestos-contaminated vermiculite.

## Figures and Tables

**Table 1:** Characteristics of study participants in 2000–2001 living in Libby, Montana, who were ≤ 18 years of age when the mine closed in 1990.

Characteristic	*n* (%)
Demographics	
Current age (years)	
10–16	495 (49.4)
17–22	304 (30.3)
23–29	204 (20.3)
Sex	
Male	429 (42.8)
Female	574 (57.2)
Smoking use	
Never	772 (77.0)
Former	73 (7.3)
Current	158 (15.8)
Residing with someone else who smokes	
Yes	508 (50.7)
No	495 (49.4)

Symptom history	
Usually have a cough^a^	
Yes	108 (10.8)
No	894 (89.2)
Coughed up phlegm that was bloody in the past year	
Yes	59 (5.9)
No	944 (94.1)
Troubled by shortness of breath when walking up a slight hill or when hurrying on level ground^a^	
Yes	145 (14.5)
No	857 (85.5)
Been told by a physician that you have a lung disease/condition	
Yes	51 (5.1)
No	952 (94.9)
Spirometry results^a^	
Normal	896 (90.5)
Obstructive	62 (6.3)
Restrictive	30 (3.0)
Mixed	2 (0.2)

a Missing data for “usually have a cough” (*n* = 1), “troubled by shortness of breath when walking up a slight hill or when hurrying on level ground” (*n* = 1), and spirometry results (*n* = 13).

**Table 2:** Age-, sex-, and tobacco-smoke exposure–adjusted associations between activities with possible exposure to asbestos-containing vermiculite and self-reported respiratory outcomes among individuals ≤ 18 years of age when the mine closed in Libby, Montana.

	Cough			Shortness of breath			Bloody phlegm			Lung disease		
Exposure	Cases^a^ (*n* = 108)	Controls^a^ (*n* = 894)	OR^b^ (95% CI^b^)	Cases^a^ (*n* = 145)	Controls^a^ (*n* = 857)	OR^b^ (95% CI^b^)	Cases^a^ (*n* = 59)	Controls^a^ (*n* = 944)	OR^b^ (95% CI^b^)	Cases^a^ (*n* = 51)	Controls^a^ (*n* = 952)	OR^b^ (95% CI^b^)
Association for all activities with potential for Libby amphibole exposure combined (no./frequency of activities^c^)												
Never	5	100	1.00	10	95	1.00	5	100	1.00	3	102	1.00
Sometimes	39	367	1.88 (0.71–5.00)	52	354	1.16 (0.55–2.44)	19	387	0.85 (0.31–2.38)	23	383	1.95 (0.57–6.71)
1–2 frequently	51	379	2.00 (0.76–5.28)	70	360	1.27 (0.61–2.63)	29	402	1.09 (0.40–2.98)	21	410	1.51 (0.43–5.24)
≥ 3 frequently	12	47	2.93 (0.93–9.25)	13	46	1.32 (0.51–3.42)	6	53	1.49 (0.41–5.43)	4	55	1.72 (0.36–8.32)

Associations with individual activities with potential for Libby amphibole exposure												
Handled vermiculite insulation												
Never	69	666	1.00	104	631	1.00	41	695	1.00	37	699	1.00
Sometimes	27	188	1.26 (0.77–2.06)	24	191	0.67 (0.41–1.09)	12	203	0.93 (0.48–1.83)	10	205	0.90 (0.44–1.86)
Frequently	11	32	3.38 (1.56–7.31)	15	28	3.28 (1.63–6.63)	6	37	2.75 (1.07–7.09)	4	39	1.85 (0.62–5.54)
Participated in recreational activities along Rainy Creek Road^d^												
Never	34	393	1.00	45	382	1.00	21	406	1.00	20	407	1.00
Sometimes	42	379	1.16 (0.70–1.90)	62	360	1.30 (0.84–2.00)	23	399	1.02 (0.55–1.91)	24	398	1.17 (0.63–2.19)
Frequently	29	115	1.97 (1.10–3.51)	36	108	1.89 (1.12–3.19)	12	132	1.28 (0.59–2.78)	6	138	0.74 (0.28–1.93)
Played at the ball fields near the expansion plant												
Never	24	235	1.00	33	226	1.00	7	252	1.00	12	247	1.00
Sometimes	38	301	1.03 (0.59–1.82)	50	289	0.92 (0.56–1.52)	21	318	2.15 (0.89–5.19)	18	321	0.99 (0.46–2.12)
Frequently	44	356	0.91 (0.52–1.59)	61	339	0.85 (0.52–1.39)	30	371	2.46 (1.04–5.79)	20	381	0.88 (0.42–1.88)
Played in or around the vermiculite piles												
Never	66	648	1.00	91	624	1.00	37	678	1.00	32	683	1.00
Sometimes	24	168	1.14 (0.67–1.93)	36	156	1.25 (0.79–1.96)	12	180	1.05 (0.52–2.10)	10	182	1.07 (0.51–2.25)
Frequently	14	65	1.46 (0.74–2.87)	14	64	0.93 (0.48–1.80)	9	70	1.85 (0.82–4.17)	7	72	1.65 (0.68–4.03)
Heated the vermiculite to make it expand/pop												
Never	70	675	1.00	109	636	1.00	44	702	1.00	35	711	1.00
Sometimes	24	181	1.26 (0.75–2.13)	23	182	0.61 (0.36–1.01)	11	194	0.81 (0.40–1.65)	10	195	0.97 (0.46–2.05)
Frequently	12	36	2.30 (1.08–4.92)	12	36	1.15 (0.55–2.39)	3	45	0.75 (0.22–2.61)	5	43	1.89 (0.68–5.28)
Participated in other activities where contact was made with vermiculite insulation, products, or ores not mentioned												
Never	65	690	1.00	104	651	1.00	45	711	1.00	39	717	1.00
Sometimes	37	168	2.29 (1.45–3.64)	37	168	1.25 (0.81–1.94)	11	194	0.84 (0.42–1.67)	10	195	0.87 (0.43–1.80)
Frequently	4	25	1.52 (0.49–4.73)	2	27	0.36 (0.08–1.62)	1	28	0.52 (0.07–4.00)	1	28	0.57 (0.07–4.39)

a Number of cases and controls do not always sum to total because of missing data on exposure.

b Adjusted for age, sex, smoking status, and living/lived with a smoker.

c “Never“ means never participating in any of the activities; “Sometimes,”participating only sometimes in any of the activities; “1–2 frequently,” participating frequently in one or two of the activities; “≥ 3 frequently,” participating frequently in three or more of the activities.

d Rainy Creek Road is the road leading to the mine.

**Table 3:** Age-, sex-, and tobacco-smoke exposure–adjusted associations between activities with possible exposure to asbestos-containing vermiculite and spirometry results among individuals ≤ 18 years of age when the mine closed in Libby, Montana.

Exposure	Cases^a^,^b^ (*n* = 94)	Controls^a^ (*n* = 896)	OR^c^ (95% CI^c^)
Association for all activities with potential for Libby amphibole exposure combined (no./frequency of activities^d^)			
Never	8	97	1.00
Sometimes	41	361	1.34 (0.60–2.96)
1–2 frequently	39	384	1.20 (0.53–2.70)
≥ 3 frequently	6	52	1.33 (0.42–4.19)

Associations with individual activities with potential for Libby amphibole exposure			
Handled vermiculite insulation			
Never	74	655	1.00
Sometimes	14	196	0.62 (0.34–1.12)
Frequently	5	38	1.10 (0.42–2.91)
Participated in recreational activities along Rainy Creek Road^e^			
Never	40	385	1.00
Sometimes	38	374	0.93 (0.58–1.49)
Frequently	15	128	1.08 (0.56–2.08)
Played at the ball fields near the expansion plant^c^			
Never	24	234	1.00
Sometimes	35	301	1.13 (0.65–1.97)
Frequently	35	357	0.94 (0.54–1.65)
Played in or around the vermiculite piles			
Never	65	642	1.00
Sometimes	17	171	0.98 (0.56–1.73)
Frequently	10	68	1.46 (0.70–3.05)
Heated the vermiculite to make it expand/pop			
Never	71	666	1.00
Sometimes	18	184	0.86 (0.49–1.51)
Frequently	5	42	1.04 (0.39–2.79)
Participated in other activities where contact was made with vermiculite insulation, products, or ores not mentioned			
Never	68	678	1.00
Sometimes	22	180	1.22 (0.73–2.04)
Frequently	1	28	0.36 (0.05–2.69)

a Number of cases and controls do not always sum to total because of missing data on exposure.

b Spirometry results were considered abnormal if they were classified as restrictive, obstructive, or mixed.

c Adjusted for age, sex, smoking status, and living/lived with a smoker.

d “Never“ means never participating in any of the activities; “Sometimes,” participating only sometimes in any of the activities; “1–2 frequently,” participating frequently in one or two of the activities; “≥ 3 frequently,” participating frequently in three or more of the activities.

e Rainy Creek Road is the road leading to the mine.
